# Supplementary material for: Observation of novel topological states in hyperbolic lattices
Source: Nat Commun. 2022 May 26;13:2937. doi: 10.1038/s41467-022-30631-x (PMC9135738; doi:10.1038/s41467-022-30631-x)
Supplement: Supplementary file 1 — Supplementary information [file 41467_2022_30631_MOESM1_ESM.pdf]

Supplementary Information: Observation of novel topological states in hyperbolic lattices

W. Zhang et al.

Supplementary note 1. The geometric property of hyperbolic lattices.

Supplementary note 2. Numerical results of the real-space Chern number with different lattice sizes and summation regions.

Supplementary note 3. Coupled model equations of the hyperbolic Chern lattice.

Supplementary note 4. Numerical results of the robust propagation of wave packets in hyperbolic Chern insulators with different sizes.

Supplementary note 5. Numerical results of the trivial hyperbolic lattice model.

Supplementary note 6. Details for the derivation of circuit eigenequations and the correspondence to the Hyperbolic Chern insulator.

Supplementary note 7. The influence of lossy effects on impedance responses of circuit networks.

Supplementary note 8. Simulation results of the propagation for a voltage packet in designed hyperbolic Chern circuits.

Supplementary note 9. Topological properties of higher-order zero modes in the deformed hyperbolic lattice.

Supplementary note 10. Details for the derivation of circuit eigenequations and the correspondence to the deformed hyperbolic lattice with higher-order zero modes.

Supplementary note 1. The geometric property of hyperbolic lattices. In this section, we introduce some basic properties of the hyperbolic lattice embedded in the Poincaré disk model and show the general method to produce the finite hyperbolic lattice {6, 4} considered in our work. The Poincaré disk $\mathbb{D} = \{z \in \mathbb{C}, |z| < 1\}$ is defined in a 2D complex plane (z=x+iy) with the limitation of |z|<1. The hyperbolic metric used in the model is defined by

$$ds^2 = (2\kappa)^2 \frac{dx^2+dy^2}{(1-|z|^2)^2}. \quad (1)$$

Here, κ is the curvature radius, which determines the value of constant negative curvature by $K = -\kappa^{-2}$. In addition, the hyperbolic distance between two points z and z' in the Poincaré disk is expressed as:

$$d(z,z') = \kappa \,\mathrm{arcosh}\left(1 + \frac{2|z-z'|^2}{(1-|z|^2)(1-|z'|^2)}\right), \quad (2)$$

which reduces to |z-z'| for $|z|$, $|z'| \ll 1$. This is because the hyperbolic metric becomes flat for $|z| \ll 1$. The boundary of the Poincaré disk is infinitely far from the interior point, and the corresponding geodesics are circular arcs perpendicular to the boundary. The isometries of the Poincaré disk are the maps that preserve the hyperbolic distance, which are fractional linear transformations expressed as:

$$z \mapsto Mz := \frac{az+b}{b^*z+a^*}, \quad a,b \in \mathbb{C}, \quad |a|^2 - |b|^2 = 1 \tag{3}$$

The map M could be written in the matrix form by

$$M = \begin{pmatrix} a & b \\ b^* & a^* \end{pmatrix}, \tag{4}$$

which corresponds to the non-abelian group $PSU(1,1)$.

For the hyperbolic lattice model, the continuum space becomes discrete. In this case, the isometries of Poincaré disks embedded with hyperbolic lattices should be the discrete subgroup of $PSU(1,1)$. The orientation-preserving (detM=+1) full space group of the hyperbolic lattice $\{p, q\}$ is called the proper triangle group

$$\Delta^+(p,q,2) =< A,B|A^p = B^q = (AB)^2 = I > \tag{5}$$

with group generators A and B being expressed as

$$A = \begin{pmatrix} e^{i\alpha/2} & 0 \\ 0 & e^{-i\alpha/2} \end{pmatrix}$$

$$B = \frac{1}{1-r_0^2}\begin{pmatrix} e^{\frac{i\beta}{2}} - r_0^2 e^{-\frac{i\beta}{2}} & r_0\left(1-e^{i\beta}\right)e^{\frac{i(\alpha-\beta)}{2}} \\ r_0\left(1-e^{-i\beta}\right)e^{-\frac{i(\alpha-\beta)}{2}} & e^{-\frac{i\beta}{2}} - r_0^2 e^{\frac{i\beta}{2}} \end{pmatrix}$$

$$r_0 = \sqrt{\frac{\cos(\frac{\pi}{p}+\frac{\pi}{q})}{\cos(\frac{\pi}{p}-\frac{\pi}{q})}}, \ \alpha = \frac{2\pi}{p}, \beta = \frac{2\pi}{q} \tag{6}$$

Here, generator A corresponds to a rotation around the center of a face, and B corresponds to a rotation around an adjacent vertex in the hyperbolic lattice. It is worth noting that the proper triangle group defined in the hyperbolic lattice possesses a similar role as space groups in the Euclidean lattice model. Based on two generators of $\Delta^+(p,q,2)$, the coordinates of all lattice sites belonging to the {6,4} hyperbolic lattice could be easily obtained. Specifically, we start with a single 6-gon and iteratively apply products of generators A and B on all produced coordinates based on the fractional linear transformations in Eq. (6). Profiles of lattice sites with different iterations (1, 2, 3 and 4) are shown in Supplementary Figure 1, where the site distribution is in the form of the {6,4} hyperbolic lattice.

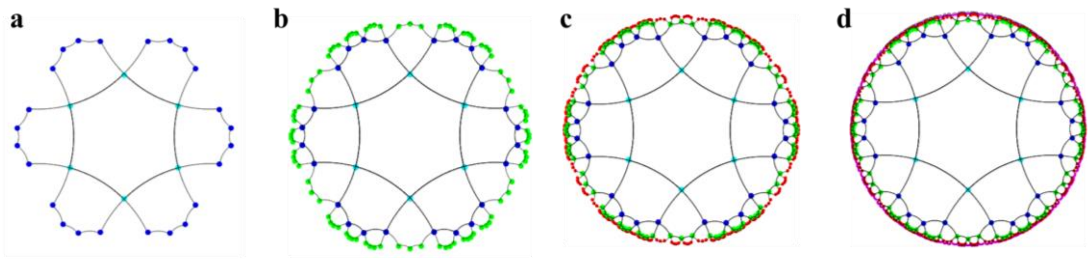

Supplementary Figure 1. Profiles of lattice sites with different iterations. Profiles of hyperbolic lattice {6, 4} with one iteration in **a**, two iterations in **b**, three iterations in **c** and four iterations in **d**.

Supplementary note 2. Numerical results of the real-space Chern number with different lattice sizes and summation regions. Previous investigations have shown that the calculated value of real-space Chern number is dependent on the position and size of the chosen summation regions I, II and III. Generally, the real space Chern number does not converge well if the lattice site in the summation region is too close to the boundary of the system (relative to the localization length of edge modes) or the summation region is too small to contain sufficient numbers of sites. To clarify the size influence on the real-space Chern number in the hyperbolic Haldane model, here, we consider a larger hyperbolic lattice with L=5 and calculate the real space Chern number with different numbers of lattice sites in the summation region. Supplementary Figures 2a-2c present the results with the site number in each summation region (I, II, III) equal to 20, 30, and 108. Moreover, the eigenspectrum of the corresponding hyperbolic lattice is also calculated, as shown in Supplementary Figure 2d, where the color bar quantifies the localization degree of associated eigenmodes on the boundary. We can see that as the number of lattice sites in each summation region increases, the calculated nontrivial Chern number gradually approach to -1.

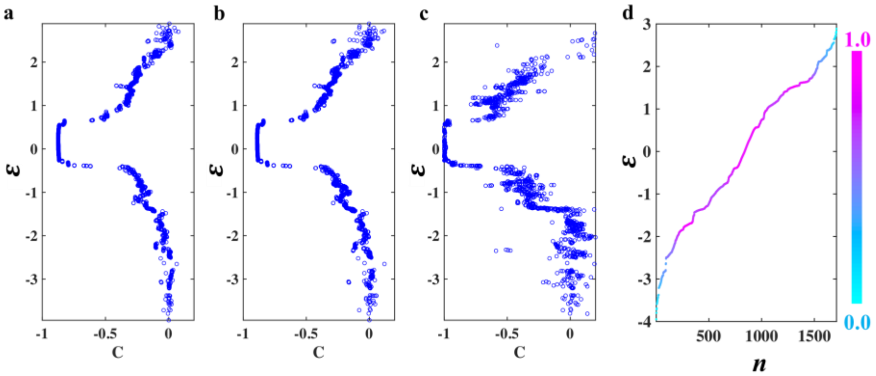

Supplementary Figure 2. The dependency of real space Chern number with the number of lattice sites. a-c. Calculated real-space Chern numbers with the site number in each summation region (A, B, C) equal to 20, 30, and 108. **d.** The eigenspectrum of the corresponding hyperbolic lattice with L=5.

Supplementary note 3. Coupled model equations of the hyperbolic Chern lattice. In this part, we construct the general coupled-mode equations in the hyperbolic Haldane model with an input port to characterize the evolution of unidirectional edge states in the hyperbolic Chern insulator. The wave function in the hyperbolic Chern insulator is expanded as:

$$\Psi(t) = \sum_i c_i(t) \tag{7}$$

where $c_i(t)$ is the probability amplitude of the wave function at site *i*. In this case, the coupled mode equation could be expressed as:

$$\frac{dc_i(t)}{dt} = -i\gamma \sum_{<i,j>} c_j(t) - i\lambda \sum_{<<i,j>>} \lambda e^{i\varphi} c_j(t) + \chi\psi_{\mathrm{in}}(\mathrm{t}) \tag{8}$$

where χ is the coupling rate of the input channel and $\psi_{\mathrm{in}}(\mathrm{t})$ is the input signal. In all calculations, we set $\chi = 0.2$. In addition, the input wave packet at the lattice site (*i*=21) is given by $\psi_{in}(t) = exp(-(t - t_0)^2/64) sin(\varepsilon_c t)\delta_{21,i}$ for simulations in Figs. 1f and 1g of the main text.

Supplementary note 4. Numerical results of the robust propagation of wave packets in hyperbolic Chern insulators with different sizes. In this part, we give numerical results of the robust propagation of wave packets in hyperbolic Chern insulators with *L*=3 and *L*=5. Supplementary Figure 3a displays the time-dependent spatial distributions of $|\boldsymbol{\psi}_{\mathrm{i}}(\mathrm{t})|$ in the hyperbolic Chern lattice with *L*=3 at t=17.8, t=44.7 and t=74.5. Positions of the excitation site and defects are marked by the green arrow and black triangles. We can see that even though the hyperbolic Chern insulator contains only three layers, the injected wave packet can still unidirectionally move along the edge and pass through defects without backscattering. Similarly, numerical results for the hyperbolic lattice with *L*=5 are presented in Supplementary Figure S3b. We note that similar phenomena with robust wave propagations on the boundary are observed.

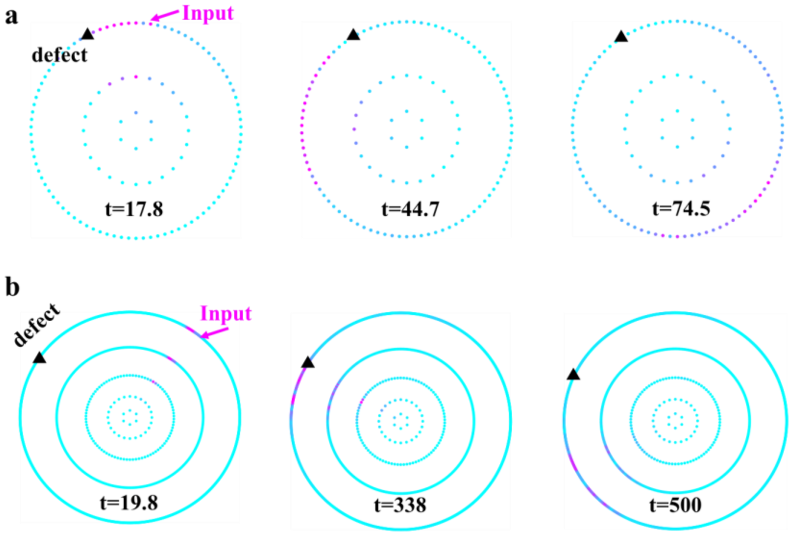

Supplementary Figure 3. Numerical results of wave propagations in the hyperbolic lattice with ***L*=5. a.** The time-dependent spatial distributions of $|\boldsymbol{\varphi}_{\mathrm{i}}(\mathrm{t})|$ at t=17.8, t=44.7 and t=74.5 for the system with *L*=3. **b.** The time-dependent spatial distributions of $|\boldsymbol{\varphi}_{\mathrm{i}}(\mathrm{t})|$ at t=19.8, t=338 and t=500 for the system with *L*=5.

Supplementary note 5. Numerical results of the trivial hyperbolic lattice model. In this part, we calculate the eigen-spectra, the corresponding real space Chern numbers, and the dynamics of injected wave packet for systems without NNN couplings $\lambda = 0$ (in Supplementary Figure 4), and with real-valued NNN couplings $\lambda = 0.2$, $\varphi = 0$ (in Supplementary Figure 5). We can see that the real-space Chern number is trivial around the zero-energy, and the one-way propagation of edge state is also absence.

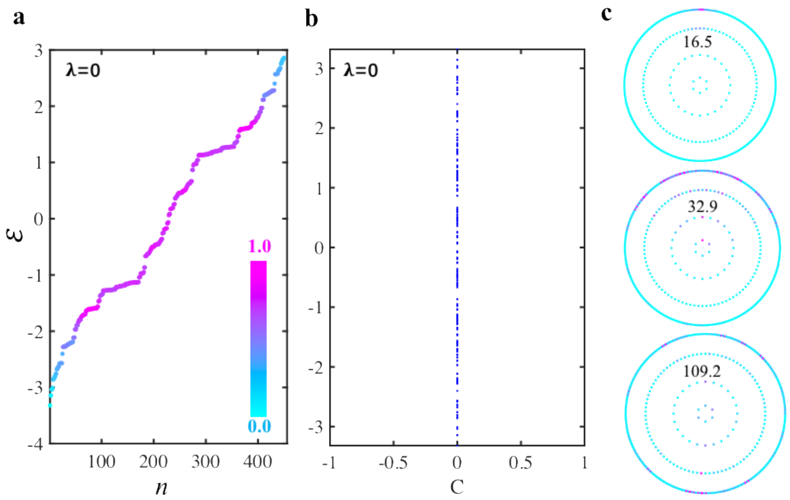

Supplementary Figure 4. Numerical results of the hyperbolic lattice without NNN couplings. a-c.

The calculated eigen-spectrum, the real space Chern numbers, and the dynamics of injected wave packet for systems with the NNN coupling being λ = 0. The color bar corresponds to the localization degree at the boundary.

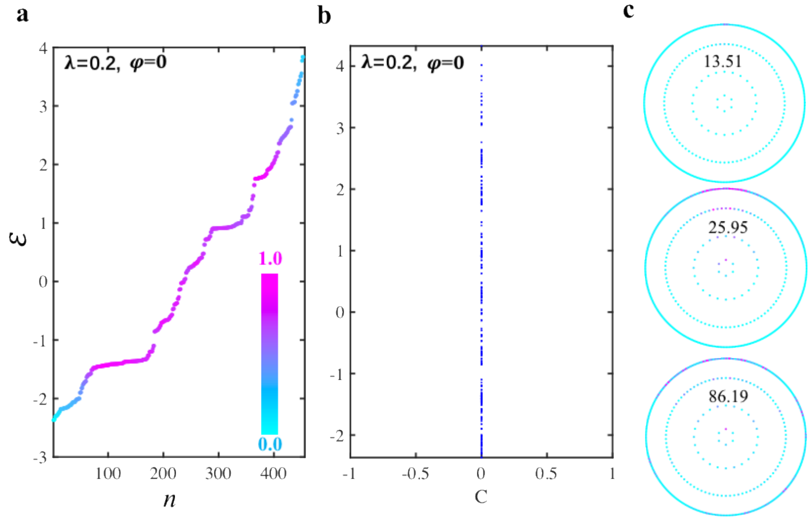

Supplementary Figure 5. Numerical results of the hyperbolic lattice with real-valued NNN couplings. a-c. The calculated eigen-spectrum, the real space Chern numbers, and the dynamics of injected wave packet for systems with parameters being λ = 0.2 and φ = 0. The color bar corresponds to the localization degree at the boundary.

Supplementary note 6. Details for the derivation of circuit eigenequations and the correspondence to the Hyperbolic Chern insulator. In this section, we give a detailed derivation of the circuit eigenequation and the correspondence between the designed hyperbolic Chern circuit and the hyperbolic Chern insulator. Here, each lattice site possesses three circuit nodes. In this case, the voltage and current at site i should be written as $V_i = [V_{i,1}, V_{i,2}, V_{i,3}]^T$ and$I_i = [I_{i,1}, I_{i,2}, I_{i,3}]^T$, respectively, and the voltage on circuit node i is in the form of $V_i e^{i\omega t}$.

First, we focus on three nodes (corresponding to a single lattice site) located in the bulk region of the hyperbolic Chern circuit. Carrying out Kirchhoff's law on three circuit nodes at i, we obtain the following equation:

$$\begin{bmatrix} I_{i,1} \\ I_{i,2} \\ I_{i,3} \end{bmatrix} = i\omega^{-1}(\omega^2 C \begin{bmatrix} 2 & -1 & -1 \\ -1 & 2 & -1 \\ -1 & -1 & 2 \end{bmatrix} \begin{bmatrix} V_{i,1} \\ V_{i,2} \\ V_{i,3} \end{bmatrix} + \sum_{<i,j>} \omega^2 C_\gamma \begin{bmatrix} V_{i,1} - V_{j,1} \\ V_{i,2} - V_{j,2} \\ V_{i,3} - V_{j,3} \end{bmatrix} +$$

$$\sum_{\ll i,j \gg \, with \, \varphi>0} \omega^2 C_\lambda \begin{bmatrix} V_{i,1} - V_{j,2} \\ V_{i,2} - V_{j,3} \\ V_{i,3} - V_{j,1} \end{bmatrix} + \sum_{\ll i,j \gg \, with \, \varphi<0} \omega^2 C_\lambda \begin{bmatrix} V_{i,1} - V_{j,3} \\ V_{i,2} - V_{j,1} \\ V_{i,3} - V_{j,2} \end{bmatrix} - \frac{1}{L_g} \begin{bmatrix} V_{i,1} \\ V_{i,2} \\ V_{i,3} \end{bmatrix}) \tag{9}$$

where C_γ and C_λ are capacitances linking nodes at i to the NN and NNN nodes at j. C is the

capacitance used for connecting circuit nodes belonging to the same site. L_g is the inductor linking the circuit nodes to the ground.

We assume that there is no external source, so that the current flowing out of the node is zero. In this case, Supplementary Eq. (9) becomes:

$$-\frac{1}{\omega^2 L_g}\begin{bmatrix}V_{i,1}\\V_{i,2}\\V_{i,3}\end{bmatrix} = C\begin{bmatrix}2 & -1 & -1\\-1 & 2 & -1\\-1 & -1 & 2\end{bmatrix}\begin{bmatrix}V_{i,1}\\V_{i,2}\\V_{i,3}\end{bmatrix} + (4C_\gamma + 8C_\lambda)\begin{bmatrix}V_{i,1}\\V_{i,2}\\V_{i,3}\end{bmatrix} - \sum_{<i,j>} C_\gamma \begin{bmatrix}V_{i,1}\\V_{i,2}\\V_{i,3}\end{bmatrix}$$

$$-\sum\nolimits_{\ll i,j\gg\ with\ \varphi>0} C_\lambda \begin{bmatrix}V_{j,2}\\V_{j,3}\\V_{j,1}\end{bmatrix} - \sum\nolimits_{\ll i,j\gg\ with\ \varphi<0} C_\lambda \begin{bmatrix}V_{j,3}\\V_{j,1}\\V_{j,2}\end{bmatrix}) \tag{10}$$

Performing the diagonalization of Supplementary Eq. (10) with a unitary transformation:

$$F = \frac{1}{\sqrt{3}}\begin{bmatrix}1 & 1 & 1\\1 & e^{i2\pi/3} & e^{i4\pi/3}\\1 & e^{i4\pi/3} & e^{i8\pi/3}\end{bmatrix}. \tag{11}$$

Supplementary Eq. (10) becomes:

$$-\frac{1}{\omega^2 L_g}\begin{bmatrix}V_{i,0}\\V_{i,\uparrow}\\V_{i,\downarrow}\end{bmatrix} = C\begin{bmatrix}0 & 0 & 0\\0 & 3 & 0\\0 & 0 & 3\end{bmatrix}\begin{bmatrix}V_{i,0}\\V_{i,\uparrow}\\V_{i,\downarrow}\end{bmatrix} + (4C_\gamma + 8C_\lambda)\begin{bmatrix}V_{i,0}\\V_{i,\uparrow}\\V_{i,\downarrow}\end{bmatrix} - \sum\nolimits_{<i,j>} C_\gamma \begin{bmatrix}V_{i,0}\\V_{i,\uparrow}\\V_{i,\downarrow}\end{bmatrix} +$$

$$(-\sum\nolimits_{\ll i,j\gg\ with\ \varphi>0} C_\lambda \begin{bmatrix}1 & 0 & 0\\0 & e^{\frac{i2\pi}{3}} & 0\\0 & 0 & e^{-\frac{i2\pi}{3}}\end{bmatrix} - \sum\nolimits_{\ll i,j\gg\ with\ \varphi<0} C_\lambda \begin{bmatrix}1 & 0 & 0\\0 & e^{-\frac{i2\pi}{3}} & 0\\0 & 0 & e^{\frac{i2\pi}{3}}\end{bmatrix})\begin{bmatrix}V_{i,0}\\V_{i,\uparrow}\\V_{i,\downarrow}\end{bmatrix} \tag{12}$$

The new basis is $V_{(0,\uparrow,\downarrow),i} = F[V_{i,1}, V_{i,2}, V_{i,3}]^T$, which are three decoupled terms and two frequency-dependent terms $V_{(\uparrow,\downarrow),i}$ acting as a pair of pseudospins $V_{\uparrow i} = V_{i,1} + V_{i,2}e^{i2\pi/3} + V_{i,3}e^{-i2\pi/3}$ and $V_{\uparrow i} = V_{i,1} + V_{i,2}e^{-i2\pi/3} + V_{i,3}e^{i2\pi/3}$. Thus, Supplementary Eq. (12) on the basis of two pseudospins can be expressed as:

$$\frac{1}{\omega^2 LC}V_{\uparrow,i} - 3 - (4C_\gamma + 8C_\lambda)/c = -\sum\nolimits_{<i,j>} C_\gamma V_{\uparrow,i} - \sum\nolimits_{\ll i,j\gg} C_\lambda e^{\pm\frac{i2\pi}{3}} V_{i,\uparrow}. \tag{13}$$

$$\frac{1}{\omega^2 LC}V_{\downarrow,i} - 3 - (4C_\gamma + 8C_\lambda)/c = -\sum\nolimits_{<i,j>} C_\gamma V_{i,\downarrow} - \sum\nolimits_{\ll i,j\gg} C_\lambda e^{\pm\frac{i2\pi}{3}} V_{i,\downarrow}. \tag{14}$$

In this case, we provide the following identification of tight-binding parameters in terms of circuit elements:

$$\gamma = \frac{C_\gamma}{C},\ \lambda = \frac{C_\lambda}{C}, \varphi = \frac{2\pi}{3},\ \varepsilon = \frac{{f_0}^2}{f^2} - 3 - \frac{4C_\gamma}{C} - \frac{8C_\lambda}{C},\ f_0 = \frac{1}{2\pi\sqrt{CL}}, \tag{15}$$

Combing Supplementary Eqs. (13-15), we note that the circuit eigenequation is consistent with the eigenequation of hyperbolic Chern insulators.

Supplementary note 7. **The influence of lossy effects on impedance responses of circuit networks.** In this part, we numerically investigated the influence of lossy effects on the impedance responses of hyperbolic circuit networks. To quantitatively estimate the loss of our circuit samples, we calculate the impedance responses (at bulk and edge nodes) of the designed hyperbolic Chern circuit with the effective

series resistances of inductance being 20 $m\Omega$, 50 $m\Omega$, 100 $m\Omega$, and 150 $m\Omega$, as shown in Supplementary Figures 6a-6d. Other circuit parameters are identical to that used in Fig. 2c of main text. It is shown that with the series resistances of inductance being increased, the impedance peak is broadening.

Similarly, we also calculate the impedance at bulk and edge nodes in the deformed hyperbolic circuit sustaining higher-order zero modes, where the effective series resistances of inductance are set as 20 $m\Omega$, 50 $m\Omega$, 100 $m\Omega$, and 150 $m\Omega$, as shown in Supplementary Figures 7a-7d. Other circuit parameters are identical to that used in Fig. 4c of main text. The loss induced broadening of impedance peaks also appears.

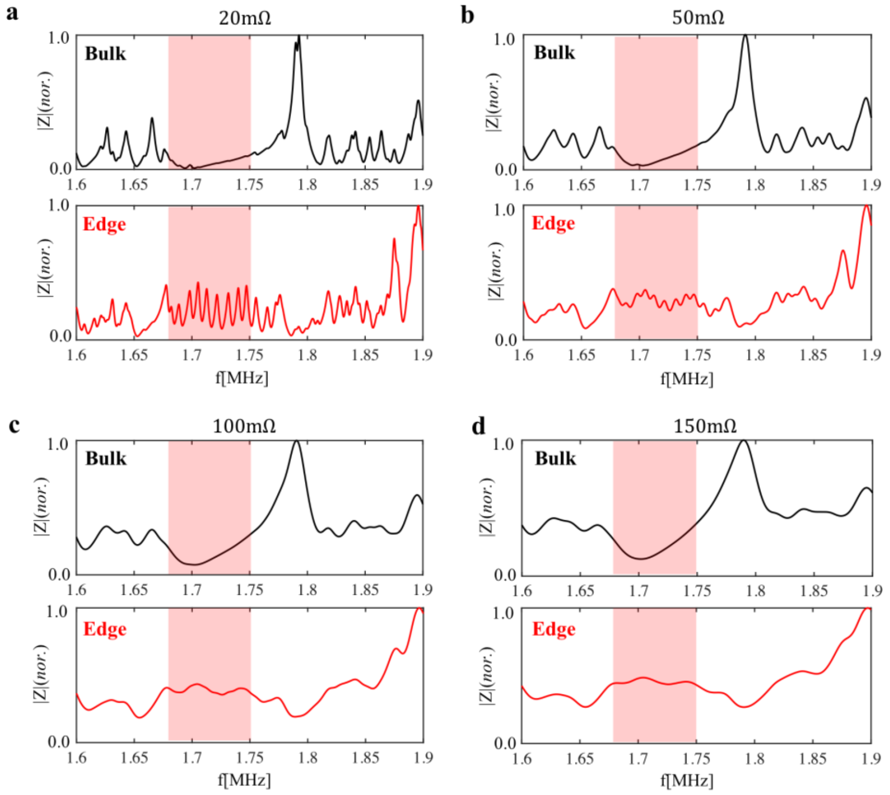

Supplementary Figure 6. Calculated impedance responses of the hyperbolic Chern circuit with different effective series resistances of inductance. a-d. The simulated impedance responses (at bulk and edge nodes) of the designed hyperbolic Chern circuit with the effective series resistances of inductance being 20 $m\Omega$, 50 $m\Omega$, 100 $m\Omega$, and 150 $m\Omega$, respectively. Other circuit parameters are identical to that used in Fig. 2c.

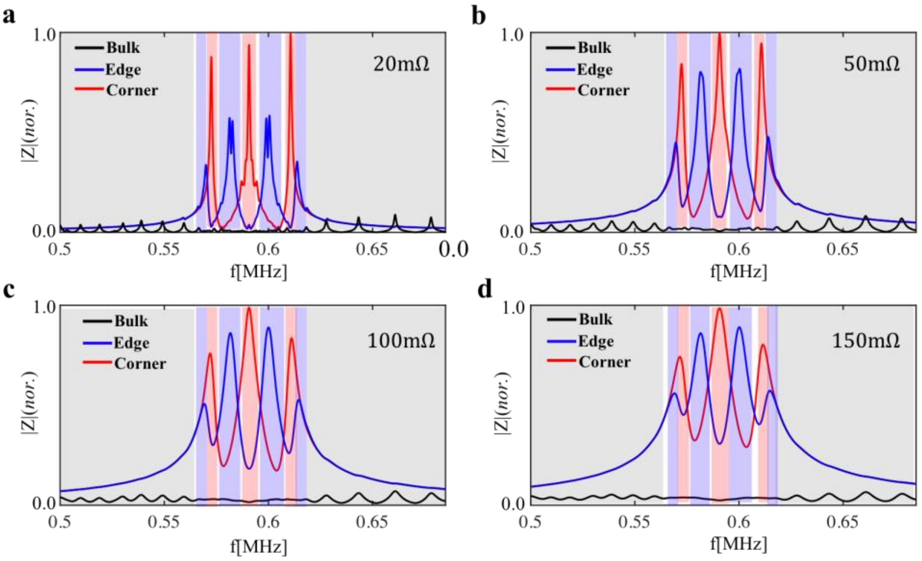

Supplementary Figure 7. Calculated impedance responses of the higher-order hyperbolic circuits with different effective series resistances of inductance. a-d. The simulated impedance responses of the designed hyperbolic circuit sustaining higher-order zero modes with the effective series resistances of inductance being 20 $m\Omega$, 50 $m\Omega$, 100 $m\Omega$, and 150 $m\Omega$, respectively. The red, blue and black lines correspond to results of corner, edge and bulk nodes. Other circuit parameters are identical to that used in Fig. 4c.

Supplementary note 8. Simulation results of the propagation for a voltage packet in designed hyperbolic Chern circuits. In this part, we calculate the voltage dynamics in the designed hyperbolic Chern circuit with L=3, where values of circuit elements are chosen as C=1 nF, C_γ=1 nF, C_λ=0.2 nF, L_g=1 uH, and C_P=5 nF. Three boundary circuit nodes are simultaneously excited as $[\mathrm{V_{i,1}}, \mathrm{V_{i,2}}, \mathrm{V_{i,3}}] = \mathrm{V(t)}[1, \exp(\mathrm{i}\frac{2\pi}{3}), \exp(-\mathrm{i}\frac{2\pi}{3})]$ with $V(\mathrm{t}) = \exp(-(\mathrm{t}-\mathrm{t_0})^2/t_d{}^2)\sin(2\pi \mathrm{f_c t})$ (t_0=70 μs, t_d=28 μs and f_c=1.706 MHz). Supplementary Figure 8a presents the calculated evolution of pseudospin $|V_{\downarrow,i}(t)|$ in the hyperbolic Chern circuit. Distributions of the voltage pseudospin at t=67us, t=94us, and t=120us are plotted in Supplementary Figures 8b-8d. It is shown that the pseudospin $|V_{\downarrow,i}(t)|$ propagates unidirectionally along the edge of the hyperbolic circuit, and no significant backscattering appears when the voltage signal passes through the defect. Furthermore, in Supplementary Figure 8e, we present time tracks of $|V_{\downarrow,i}(t)|^2$ in the defect-free circuit at two nodes, which are counterclockwise and clockwise to the excitation point with equal distances, respectively. It is clearly shown that only the counterclockwise circuit node possesses a significant peak in the time-domain, indicating that the voltage packet propagates counterclockwise along the edge of hyperbolic Chern circuit. Then, we calculate the voltage signal at

these two nodes with the existence of a defect, as shown in Supplementary Figure 8f. It is shown that the defect nearly has no influence on the voltage signals at these two nodes, manifesting the robust one-way propagation of edge states.

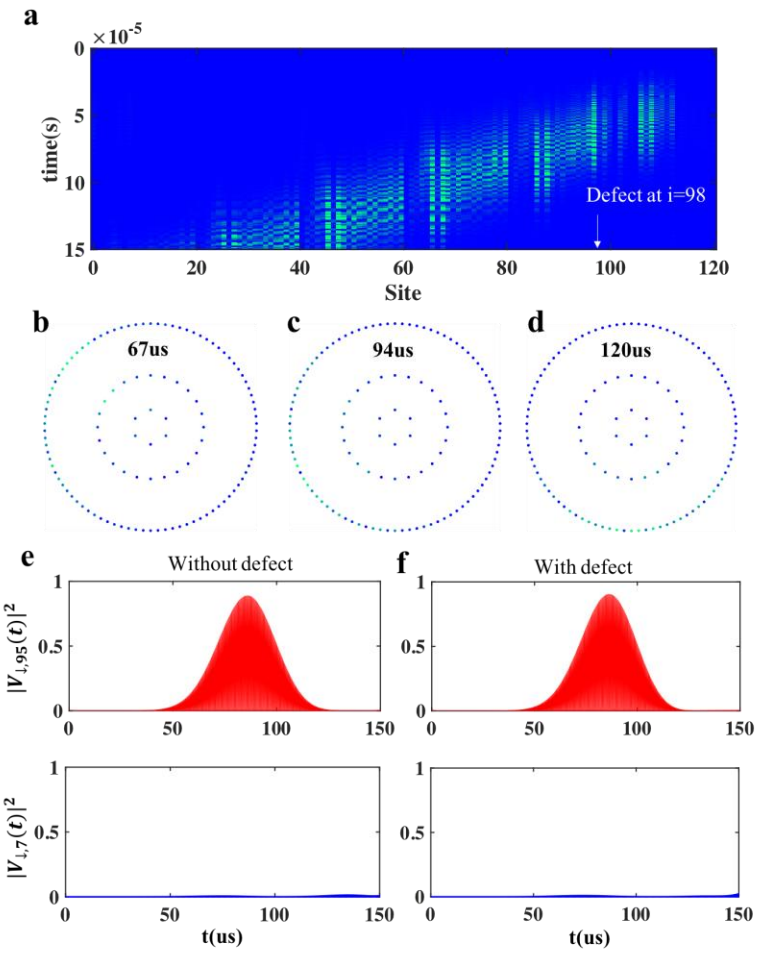

Supplementary Figure 8. Simulation results of the propagation for a voltage packet in designed hyperbolic Chern circuits. a. The calculated evolution of pseudospin $|V_{\downarrow,i}(t)|$ in the hyperbolic Chern circuit. b-d. Distributions of the voltage pseudospin at t=67us, t=94us, and t=120us. The calculated time tracks of the voltage pseudospin at circuit nodes along the counterclockwise and clockwise directions with respect to the excitation node without defects for **e** and with defects for **f.**

Supplementary note 9. Topological properties of higher-order zero modes in the deformed hyperbolic lattice. In this part, we demonstrate the topological properties of our proposed hyperbolic zero-energy corner states from three aspects.

Firstly, we focus on the topological phase transition induced by the unbalanced site coupling in the deformed hyperbolic lattice. As shown in Supplementary Figures 9a-9c, we plot the calculated eigen-

spectra of the deformed hyperbolic lattice with the ratio of γ_1/γ_2 being 10, 1 and 0.1, respectively. The color bar corresponds to the participation ratio (PR) of each eigen-mode. We set L=6, where the influence of finite size effect could be neglected. It is clearly shown that when the coupling strength in the outermost layer is the larger one (γ_1/γ_2=10), the edge states are gapped around the zero energy, and there is no midgap corner state. In such a case, this bandgap is a trivial gap. The topological phase transition could appear with closing and reopening of the bandgap. It is shown that the gap is closing (around the zero-energy) with balanced values of γ_1 and γ_2 (γ_1/γ_2=1), corresponding to the original {6,4} hyperbolic lattice. By further decreasing the ratio to the case with a smaller coupling strength in the outermost layer (γ_1/γ_2=0.1), the gap of edge states is reopened, and the midgap corner modes appear. Such a gap closing and reopening phenomenon associated with the appearance of midgap corner states is a convenient evidence for the topological phase transition.

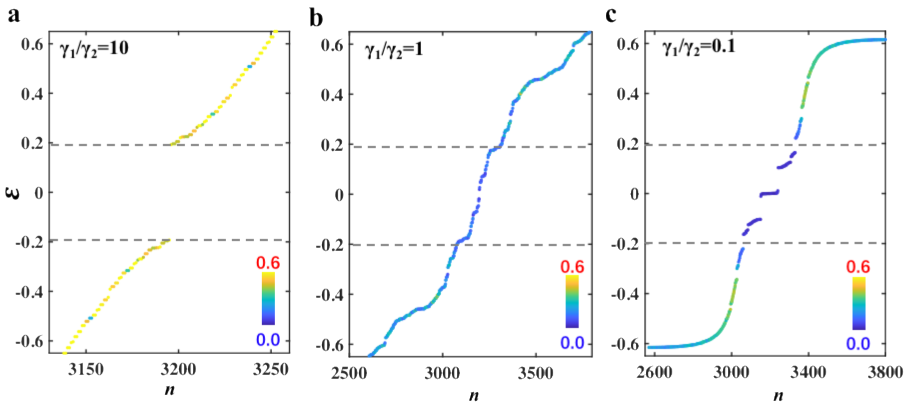

Supplementary Figure 9. The calculated eigen-spectra of the deformed hyperbolic lattice with different ratios of γ_1/γ_2. a-c. The calculated eigen-spectra of the deformed hyperbolic lattice with the ratio between γ_1 and γ_2 being 10, 1 and 0.1, respectively. Here, we set *L*=6.

Next, we show that the topological phase transition appearing in the deformed hyperbolic lattice is similar to the Euclidean counterpart of the C6-symmetric higher-order topological insulator. To clearly prove this similarity, we consider the C6-symmetric higher-order topological insulator, as shown in Supplementary Figure 10a, where the different values of intra-cell (γ_1) and inter-cell (γ_2) couplings exist. In the following calculations, the finite C6-symmetric {6,3} lattice containing 37 units is considered. As shown in Supplementary Figures 10b-10d, we calculate the eigen-spectra of the system with the ratio between γ_1 and γ_2 being 10, 1 and 0.1, respectively. We can see that, by tuning the ratio of intra- and inter-cell couplings, the topological phase transition accompanied with the closing and reopening of bandgap happens. And, the midgap corner state could appear in the non-trivial bandgap. This phenomenon is identical to the above discussed hyperbolic topological phase transition. In this case, due to the same

topological phase transition and identical symmetries (the C_6 rotation, the time reversal, and chiral symmetries), we deduce that midgap higher-order zero modes in deformed hyperbolic lattices possess similar characteristics to the filling anomaly induced 0D corner states in C6-symmetric higher-order topological crystalline insulators.

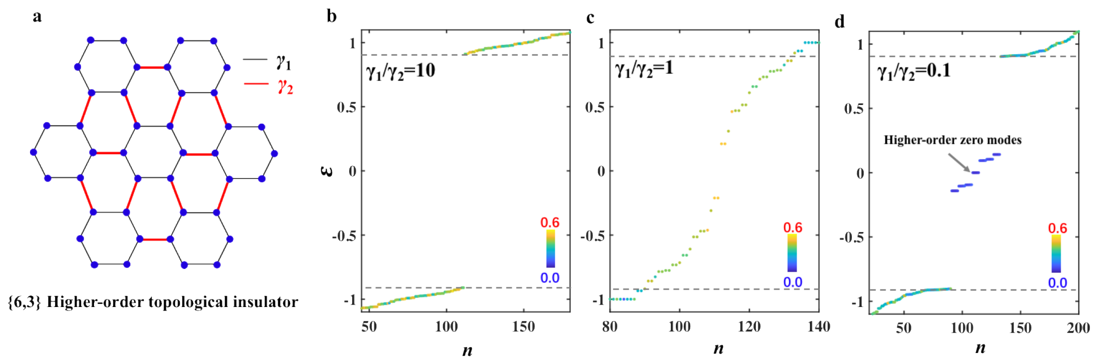

Supplementary Figure 10. The calculated eigen-spectra of the C_6-symmetric Euclidean lattice with different ratios of γ_1/γ_2. a. The schematic diagram of the C_6-symmetric higher-order topological insulator, where the intra-cell (γ_1) and inter-cell (γ_2) couplings exist. **b-d**. The eigen-spectra of the C6-symmetric lattice with the ratio between γ_1 and γ_2 being 10, 1 and 0.1, respectively. Here, in the calculation, the finite C6-symmetric lattice contains 37 units.

Finally, to further differentiate topological corner states from edge states or trivial corner states, we investigate the robustness of the midgap zero modes in the deformed hyperbolic lattice. Here, we introduce a little disorder to the onsite potential [-W, W] at all bulk sites and edge sites, and keep the onsite potential of corner sites unchanged. Other parameters are set as γ_1=1, γ_2=10 and L=6. Supplementary Figures 11a and 11b plot the calculated eigen-spectra with W being 0.1 and 0.2, respectively. The colormap corresponds to the PR. We can see that the random onsite potential could alter the eigen-energies and localizations of trivial edge states. While, the midgap zero modes are always fixed, and the corresponding mode distribution, as shown in Supplementary Figure 11c with W=0.2, also keeps the same. These numerical results manifest the robustness of hyperbolic zero modes. Because, disorders should break the enhanced energy degeneracies and interference effects in the trivial hyperbolic lattice, which makes the robust property cannot exist in the interference-induced trivial edge and corner states in hyperbolic lattices sustaining flat bands.

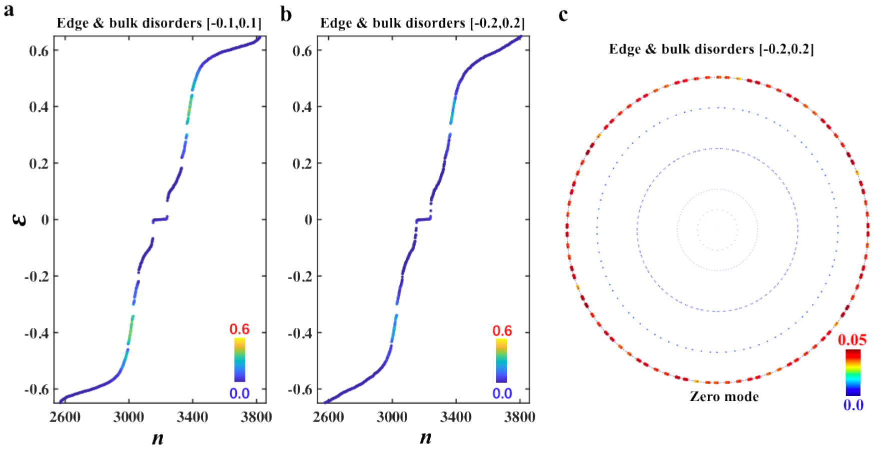

Supplementary Figure 11. The calculated eigen-spectra with different disorder strengths. a and **b**. The calculated eigen-spectra with disorder strength being W=0.1 and W=0.2, respectively. Other parameters are set as γ_1=1, γ_2=10 and L=6. **c**. The zero-mode distribution with W=0.2.

From above discussions on the topological phase transitions and robustness of zero-energy modes, we think that these hyperbolic zero-energy modes possess the same properties of their Euclidean counterparts, which results from the filling anomaly in higher-order topological crystalline insulators protected by the C6 symmetry.

Supplementary note 10. Details for the derivation of circuit eigenequations and the correspondence to the deformed hyperbolic lattice with higher-order zero modes. In this part, we give a detailed derivation of the eigenequation for the higher-order hyperbolic circuit and show the correspondence between the designed circuit lattice and the deformed hyperbolic lattice. Each bulk circuit node is connected with four adjacent nodes, where two of them are connected by C_1 and others are connected by C_2. Moreover, each circuit node is grounded by an inductor (L_{gc}). Carrying out Kirchhoff's law on the bulk circuit node, we obtain the following equation:

$$I_i = i\omega^{-1}[-\frac{1}{L_{gc}}V_i + \sum_j \omega^2 C_1(V_i - V_j) + \sum_{j'} \omega^2 C_2(V_i - V_{j'})] \tag{16}$$

where I_i and V_i are the net current and voltage at circuit node i, respectively, and j and j' mark circuit nodes that are connected with node i with C_1 and C_2, respectively.

We assume that there is no external source, so that the current flowing out of the node is zero. In this case, the Supplementary Eq. (16) becomes:

$$(\frac{f_{0c}^{\ 2}}{f^2} - 2C_1/C_2 - 2)V_i = -\sum_j (C_1/C_2)V_j - \sum_{j'} V_j' \tag{17}$$

with $f_{0c} = 1/2\pi\sqrt{C_2 L_{gc}}$.

For the edge node, there are only two NN nodes coupled with edge nodes through C_1. In this case,

we should additionally add two grounding capacitors C_2 to ensure the same resonance frequency as bulk nodes. In this case, we obtain the following circuit equation:

$$I_i = i\omega^{-1}[-\frac{1}{L_{gc}}V_i + \sum_j \omega^2 C_1\left(V_i - V_j\right) + 2\omega^2 C_2 V_i]. \quad (18)$$

Assuming there is no external source, the Supplementary Eq. (18) becomes:

$$(\frac{{f_{0c}}^2}{f^2} - 2C_1/C_2 - 2)V_i = -\sum_j (C_1/C_2)V_j \quad (19)$$

Based on the circuit eigenequation of both bulk and edge nodes, the tight-binding parameters in terms of circuit elements should be expressed as:

$$\gamma_1 = (C_1/C_2),\ \ \gamma_2 = -1,\ \varepsilon = \frac{{f_{0c}}^2}{f^2} - 2 - 2C_1/C_2, \quad (20)$$

In this case, we note that the circuit eigenequation is consistent with the eigenequation of deformed hyperbolic lattices.
